# In Situ Decoration of ZnSnO_3_ Nanosheets on the Surface of Hollow Zn_2_SnO_4_ Octahedrons for Enhanced Solar Energy Application

**DOI:** 10.3390/nano12122124

**Published:** 2022-06-20

**Authors:** Zhengdao Li, Kecheng Liu, Ruixue Sun, Chuanyun Yang, Xiaodi Liu

**Affiliations:** 1 Chemistry and Pharmaceutical Engineering College, Nanyang Normal University, Nanyang 473061, China; liukecheng2006@126.com (K.L.); ruixuesun@126.com (R.S.); ycy_chemistry@163.com (C.Y.); 2 Engineering Technology-Research Center of Henan Province for Solar Catalysis, Nanyang Normal University, Nanyang 473061, China

**Keywords:** ZnSnO_3_/Zn_2_SnO_4_, hierarchical hollow octahedron, dye-sensitized solar cells, simultaneous removal, photocatalytic activity

## Abstract

Hierarchical ZnSnO_3_/Zn_2_SnO_4_ porous hollow octahedrons were constructed using the method of combining the acid etching process with the in situ decoration technique for photovoltaic and photocatalytic applications. The composite was used as photoanode of the dye-sensitized solar cells (DSSCs), an overall 4.31% photovoltaic conversion efficiency was obtained, nearly a 73.1% improvement over the DSSCs that used Zn_2_SnO_4_ solid octahedrons. The composite was also determined to be a high-performance photocatalyst for the removal of heavy metal ion Cr (VI) and antibiotic ciprofloxacin (CIP) in single and co-existing systems under simulated sunlight irradiation. It was remarkable that the composite displayed good reusability and stability in a co-existing system, and the simultaneous removal performance could be restored by a simple acid treatment. These improvements of solar energy utilization were ascribed to the synergetic effect of the hierarchical porous hollow morphology, the introduction of ZnSnO_3_ nanosheets, and the heterojunction formed between ZnSnO_3_ and Zn_2_SnO_4_, which could improve light harvesting capacity, expedite electron transport and charge-separation efficiencies.

## 1. Introduction

Synthesis of ternary metal oxides with controllable sizes and shapes has been an active research field in the past two decades, because of their size- and shape- dependent physical, chemical, optical, electronic, and catalytic properties [1,2,3]. In particular, the two zinc stannates, Zn_2_SnO_4_ and ZnSnO_3_, have caused considerable attention due to their wide applications in dye-sensitized solar cells (DSSCs) [4,5], gas sensors [6,7], lithium-ion batteries [8,9], and photocatalysts [10,11]. Zn_2_SnO_4_ and ZnSnO_3_ are both photovoltaic and photocatalytic materials with band gaps of about 3.6 eV and 3.2 eV, and the Femi energy level of ZnSnO_3_ is higher than that of Zn_2_SnO_4_ [12,13]. When ZnSnO_3_ is coupled with Zn_2_SnO_4_, the heterojunction would form at the interface between the two ternary metal-oxide semiconductors, and the electrons would transfer from the conduction band of ZnSnO_3_ to that of Zn_2_SnO_4_ until the system reaches equilibrium of Fermi energy level [13]. This process will promote the separation of photogenerated electron–hole pairs and thus increase the performance of the DSSCs and photocatalysts. Therefore, the composite consisting of Zn_2_SnO_4_ and ZnSnO_3_ has been successfully synthesized to improve device performance [13]. For example, the ZnSnO_3_/Zn_2_SnO_4_ acted as photocatalyst for degradation of Intracron Blue, and achieved high performance under UV illumination due to lower recombination of electron-hole pairs [14]. The mixed phases of ZnSnO_3_ and Zn_2_SnO_4_ were used as photoanodes in DSSCs, and it was found the ferroelectric polarization would aid in obtaining relatively high fill factors and open-circuit voltage values in the devices [15]. 

The published research studies have demonstrated that the three-dimensional hierarchical architectures assembled from the one- or two- dimensional nanostructured building blocks are an interesting class of materials with an abundant variety of tunable physicochemical properties [16]. Hierarchical porous hollow microstructures composed of single-crystalline two-dimensional nanosheets can provide low material density, good surface permeability, high surface area and electron transport rate, as well as high light-harvesting efficiency for application areas of catalysis and photovoltaic devices [17,18]. In this structure system, the material with micrometer-sized diameters can act as light scattering centers to increase the light harvesting of the photoanodes and photocatalysts, which is one of the crucial factors of improving the photovoltaic conversion efficiency of DSSCs and photocatalytic activity [19]. The multiple-reflection inside hollow interior can provide more opportunities for the photoanodes and photocatalysts to trap the incident light for an enhanced light utilization [20,21]. The porous hollow structures possess a large surface area, and to a great extent enhance the mass transportation and the diffusion of liquid reactant in the active layers, meanwhile, the high porosity can provide rich channels with the sizes from nanometer to micron for the surface accessibility [22]. In addition, nanosheets can offer direct pathways for photogenerated electrons, which will result in enhanced electron transport rates [23]. Hence, the hierarchical porous hollow semiconductors were acknowledged as the ideal candidates for the high-performance DSSCs and photocatalysts. However, as far as we know now, there was scarcely any previous research on hierarchical porous hollow ZnSnO_3_/Zn_2_SnO_4_ with the view of the photovoltaic and photocatalysis fields simultaneously.

**Scheme 1:**
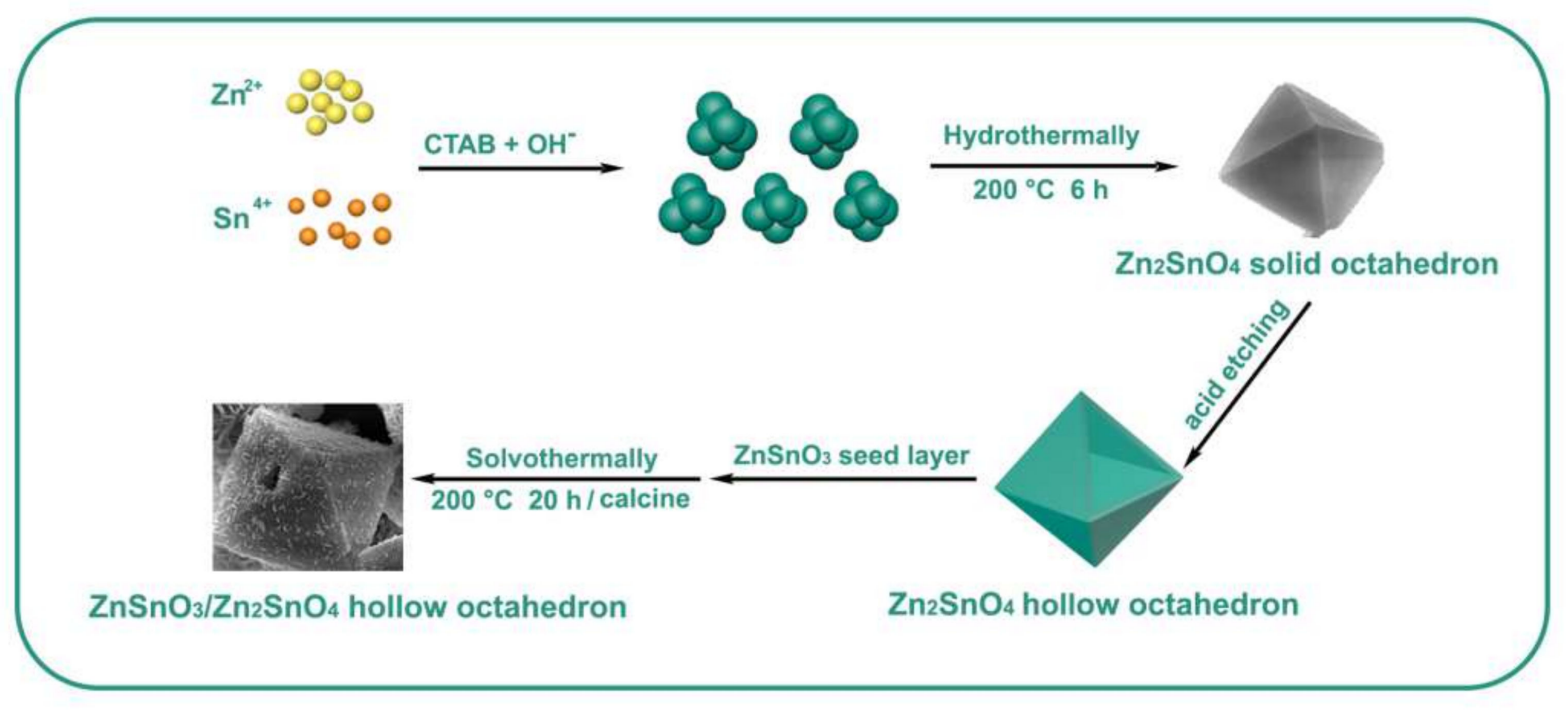
Schematic illustration for the formation of the three samples.

In our recent work, well-defined octahedral Zn_2_SnO_4_ was synthesized through a chemical solution route, and these Zn_2_SnO_4_ micro-crystals were converted to hollow octahedrons by an acid etching process. Whereafter, by means of an in situ growth method, ZnSnO_3_ nanosheets were intentionally introduced on the surface of hollow Zn_2_SnO_4_ octahedrons to construct the hierarchical porous hollow ZnSnO_3_/Zn_2_SnO_4_ octahedrons [12] (Scheme 1). In this paper, the as-prepared hollow ZnSnO_3_/Zn_2_SnO_4_ octahedrons were used as photoanodes of DSSCs to evaluate their photovoltaic performance. Meanwhile, the photocatalytic properties of the composite under simulated sunlight irradiation were evaluated by the removal of antibiotic ciprofloxacin (CIP) and heavy metal ion Cr(VI) in single and co-existing systems. Significantly, the ZnSnO_3_/Zn_2_SnO_4_ composite consisting of the two ternary metal oxides presented distinct advantages and had a higher photovoltaic conversion efficiency and better removal performance than solid and hollow Zn_2_SnO_4_ octahedrons. In addition, the morphology, crystal structure, optical properties and photocatalytic stability of the composite were investigated via various characterization methods, and the causes of enhanced photovoltaic and photocatalytic performance were proposed and discussed in detail. 

## 2. Materials and Methods

### 2.1. Materials Synthesis

All chemicals were purchased from Shanghai Aladdin Bio-Chem Technology Co. Ltd. (Shanghai, China) and used without purification. Zn_2_SnO_4_ solid octahedron (S_1_), Zn_2_SnO_4_ hollow octahedron (S_2_), hierarchical ZnSnO_3_/Zn_2_SnO_4_ hollow octahedron (S_3_), and ZnSnO_3_ nanosheets were prepared according to a reported procedure in our previous paper [12], and more details are described in the “Materials Synthesis” section in the Supplementary Materials.

### 2.2. Materials Characterization

The scanning electron microscopy (SEM, FEI NOVA NanoSEM230) and transmission electron microscopy (TEM, JEOL 3010) images were collected to obtain the morphology information of the samples. The compositions were analyzed by X-ray diffraction (XRD) (Rigaku, Tokyo, Japan) with Cu Ka radiation (λ = 1.54056 Å). 

### 2.3. Preparation of the Electrode

An amount of 1 g of as-prepared sample powder and a certain amount of absolute ethanol were put into a mortar and stirred for 30 min. An amount of 4 g of the ethanol solution of two ethyl celluloses and 2 g of anhydrous terpineol were added, and the homogenization was performed using a sonicator (Vibra cell 72408, Bioblock Scientific, Wasserbad, Germany). The contents were concentrated by evaporator with 120 mbar at 38 °C and the pastes were finalized. Fluorine-doped tin oxide (FTO) conductive glass was cleaned with dilute acid and alkali solution, ethanol and deionized water several times in the ultrasonic cleaner. A doctor-blade technique was employed to coat paste onto the surface of FTO. The film was dried at 125 °C, annealed at 450 °C for 30 min and sintered at 500 °C for 15 min in the air to form the working electrodes. Typically, to improve the connection among ZnSnO_3_/Zn_2_SnO_4_ hollow octahedrons, the as-prepared working electrodes were immersed into ZnSnO_3_ sol for 30 min, then sintered at 500 °C for another 30 min.

A magnetron sputter platinum mirror acted as the counter electrode. Dye sensitization was accomplished by immersing a film in 0.3 mM dye solution (solvent mixture of tert-butyl alcohol and acetonitrile in a volume ratio of 1:1) at 80 °C over *a set length of time*. Here, the dye used in the DSSCs was cis-bis (isothiocyanato) bis (2,2′-bipyridyl-4,4′-dicarboxylato) ruthenium-(II) bis-tetrabutylammonium (also known as N719) [24]. The electrolyte contained 0.5 M tert-butylpyridine, 0.5 M LiI and 0.05M I_2_ in acetonitrile. The FTO substrate, film, and counter electrode constituted a sandwich-like cell structure (Figure S1).

### 2.4. Photovoltaic Characterization

Electrochemical impedance spectroscopic (EIS) measurements were carried out with a PAR2273 workstation (Princeton Applied Research, Oak Ridge, TN, USA). The current–voltage (J-V) characteristics were measured with an active area of 0.16 cm^2^ on an Oriel 92251A-1000 on a Keithley 236 source measurement unit. An Oriel 92251A-1000 sunlight simulator was used as the light source (AM1.5, 100 mW cm^−y^). Intensity-modulated photocurrent/photovoltage spectra (IMPS/IMVS) were recorded on an electrochemical workstation (Zahner, Zennium, Kansas City, MO, USA). The incident photon-to-current efficiency (IPCE) was investigated on a PEC-S20 instrument (Peccell, Yokohama-shi, Japan). The film thickness was measured by a computerized profilometer (Dektak 3, Ulvac, Chigasaki, Japan). The dye-loading amount of photoanodes was ascertained by desorbing the dye into 10 mL of 0.1 M NaOH water solution and quantified by measuring N719 optical adsorption peak intensity. 

### 2.5. Photocatalytic Measurements

The photocatalytic activities of the different photocatalysts were evaluated by the removal of CIP and Cr(VI) using a 500 W xenon lamp as an irradiation source. Photocatalyst (75 mg) was added into 150 mL of CIP (10 mg/L) or/and K_2_Cr_2_O_7_ (50 mg/L) single contaminant or contaminant mixture. To achieve adsorption-desorption equilibrium, the suspension was continuously stirred for 24 h at room temperature in the dark. During the catalytic reaction, at the given intervals, 5 mL of suspension was collected and centrifuged with 4000× *g* rpm for 5 min to remove the photocatalyst. The concentration of Cr(VI) was estimated using the standard diphenylcarbazide analytical method at 540 nm with a UV-Vis spectrometer. The contents of CIP were analyzed by detecting the values of feature absorbance of 277 nm on a UV–Vis spectrometer. To study the reusable ability of the photocatalysts, the used samples were collected and washed with deionized water to remove the residual materials, followed by drying at 60 °C, and then the cycling experiments were conducted at optimal operating conditions.

## 3. Results and Discussion

### 3.1. Structure Characterization

The SEM image in the Figure S2a clearly demonstrated that the typical S_1_ showed the octahedral morphology with an average size of ~1.2 μm and possessed smooth surfaces. After a simple acid etching process, these Zn_2_SnO_4_ octahedrons still maintained their original size and shape whilst also having rough surfaces (Figure S2b). As with design, S_1_ converted into hollow structure, which could be confirmed by the TEM image of Figure S2c, to gain the sample S_2_. As can be seen from the Figure S2d, the ZnSnO_3_/Zn_2_SnO_4_ composite remained octahedron-like in structure, and the nanosheets of ~50 nm length and ~15 nm thickness distributed on each facet of the octahedron to form a hierarchical structure. Additionally, the TEM image revealed that the hollow structure was not damaged through the solvothermal process of ZnSnO_3_ nanosheet growth (Figure S2e).

Figure S3 presented the XRD spectra of the three samples. All the diffraction peaks of S_1_ and S_2_ could be assigned to the cubic phase Zn_2_SnO_4_ (JCPDS: 24-1470), this result illustrated that no change of composition and crystal form transformation emerged during the acid etching process. For the cases of S_3_, the additional XRD peaks characteristic of ZnSnO_3_ (JCPDS No. 28-1486) confirmed that S_3_ was ZnSnO_3_/Zn_2_SnO_4_ composites.

**Figure 1:**
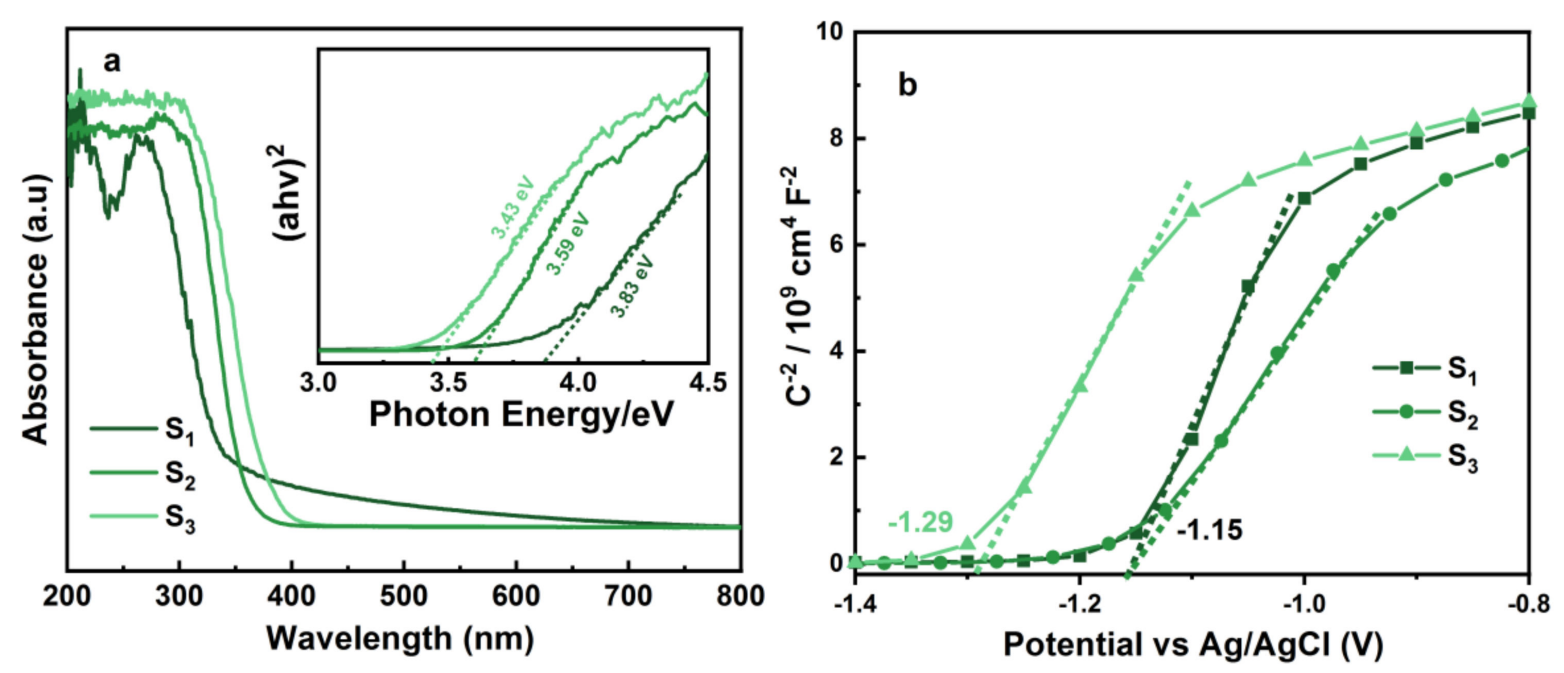
(**a**) UV−_vis absorption spectra; (**b**) Mott−Schottky plots of the samples; Inset of (**a**): the corresponding Tauc’s curves.

**Scheme 2:**
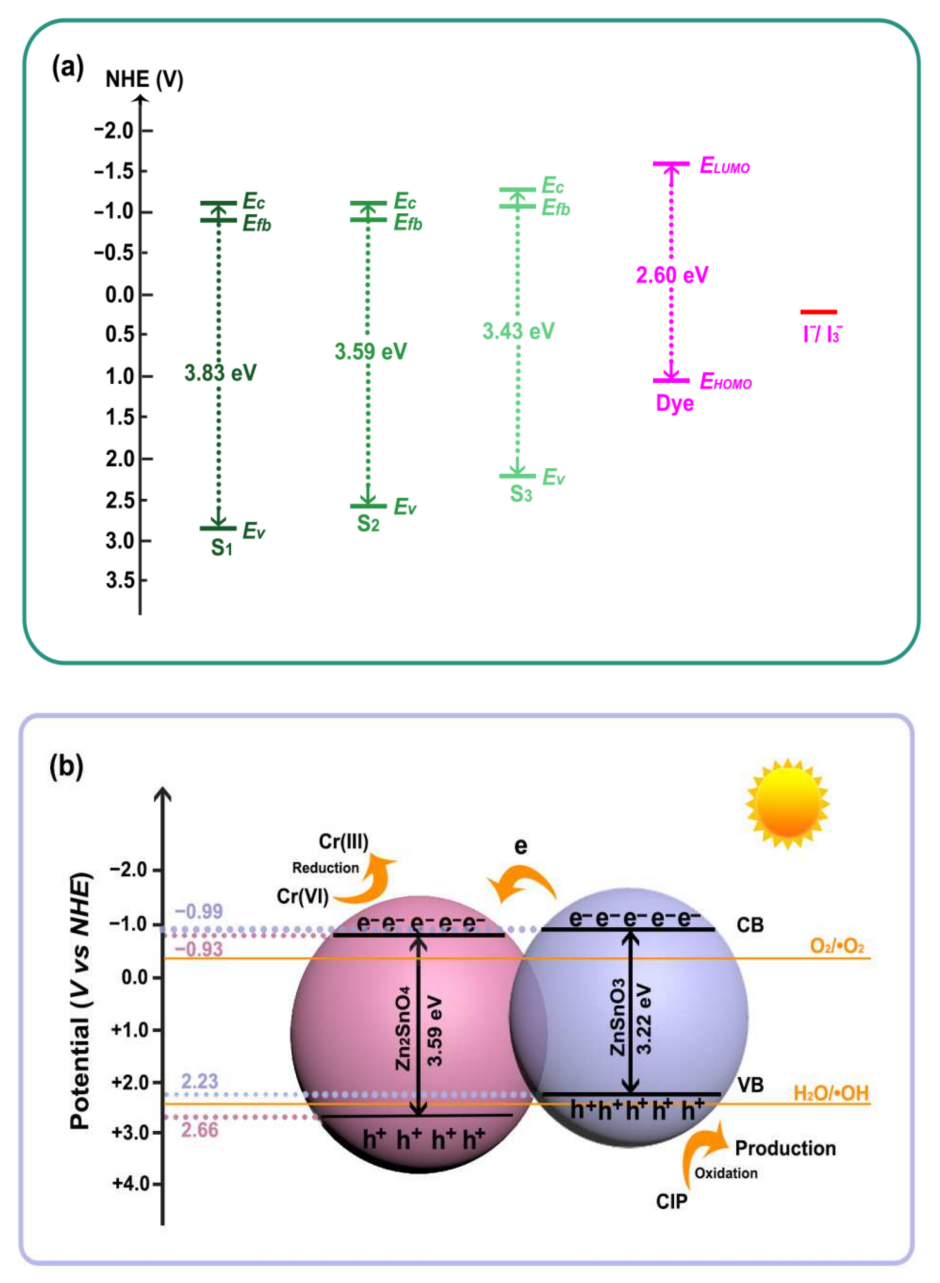
(**a**) Energy band construction of three samples; (**b**) Schematic diagram of the band structure and charge separation in ZnSnO_3_/Zn_2_SnO_4_ composite.

To construct an energy band diagram for S_1_, S_2_ and S_3_, the band gap energies (E_g_) and flat-band potentials (E_fb_) of the three samples should be determined. As shown in Figure 1a, the wavelength threshold of the pristine solid and hollow Zn_2_SnO_4_ octahedrons located at 340 nm and 360 nm, separately, and ZnSnO_3_/Zn_2_SnO_4_ hollow octahedrons displayed the red-shifted light absorption in comparison with pure Zn_2_SnO_4_, which was helpful to extend the light absorption. The E_g_ was estimated according to the Kubelka-Munk function equation to be around 3.83 eV for S_1_, 3.59 eV for S_2_ and 3.43 eV for S_3_ (the inset of Figure 1a). The positive slopes for Mott–Schottky plots indicated the characteristics of n-type semiconductors for the three samples, and the E_fb_ values were calculated to be −1.15, −1.15 and −1.29 V for S_1_, S_2_ and S_3_ (vs. Ag/AgCl, pH = 7), respectively, from the tangential intercept of the curves (Figure 1b). Then, the conduction band (CB) potentials of S_1_, S_2_ and S_3_ could be determined to be −0.93, −0.93 and −1.07 V (vs NHE, pH = 7), respectively, from the formula (Equation (1)) [25]:

(1) $$E_{\mathrm{NHE}}=E_{\mathrm{Ag}/\mathrm{AgCl}}-{\;E}^{\theta }+0.059\;\mathrm{pH}$$

where E^θ^ and E_Ag/AgCl_ represent standard electrode potentials and the Ag/AgCl electrode potential at pH = 7 (0.197 V), respectively. According to the formula (E_VB_ = E_CB_ + E_g_), the valence band (VB) potentials of S_1_, S_2_ and S_3_ were calculated to be 2.90, 2.66 and 2.36 V, respectively. Based on the E_fb_, E_CB_ and E_VB_ values of the three samples (Table S1), their energy band constructions were depicted in Scheme 2a. For the three materials, the bottom of CB and the top of VB were lower than lowest unoccupied molecular orbital (LUMO, −1.5 eV vs. NHE) and the highest occupied molecular orbital (HOMO, +1.1 eV vs. NHE) energy levels of N719 dye, respectively, and match well with the redox couple in the electrolyte, ensuring the operation of the DSSCs [26].

### 3.2. Photovoltaic Characteristics of Zn_2_SnO_4_ and ZnSnO_3_/Zn_2_SnO_4_

#### 3.2.1. Photovoltaic Characteristics of Zn_2_SnO_4_

Four DSSCs based on the Zn_2_SnO_4_ solid octahedrons photoanodes with the same thickness of ~10 µm were sensitized with various sensitization durations to examine the optimum immersion time in N719 dye solution (Table S2). Under 1 sun AM 1.5 illumination, the short-circuit current (*J*_sc_) increased with the sensitization time in the initial 6 h, while the open-circuit voltage (*V*_oc_) and fill factors (FF) remained stable, then the photovoltaic conversion efficiency (*η*) reached the maximum 2.35% at 6 h. However, a decrease in the FF, *J*_sc_ and *η* were observed at 8 h later probably because of the formation of aggregates, as in the case of Zn_2_SnO_4_ [27]. Reduced *V*_oc_ was largely due to the formation of Zn^2+^/dye complex. The immersion time was longer, a complex layer could be observed and formed a thick covering layer, which was therefore inactive for electron injection [28]. These results suggested that the optimal sensitization time was six hours in the acidic dye molecules. To explore the effect of film thickness on *η* of DSSCs, solid Zn_2_SnO_4_ films with the different thicknesses of 6, 9, 12, 15 and 18 μm were prepared and the detailed photovoltaic parameters of DSSCs based on these photoelectrodes are summarized in Table S3. In effect, when the thickness varied from 6 μm to 12 μm, the thicker film could adsorb much more dye to enhance the light harvesting ability of the photoanode, and then *J*_sc_ significantly increased from 4.17 to 5.65 mA cm^−2^. As the film thickness increased continuously to 15 μm or thicker, the more trapping sites and electron recombination centers would generate, so the more serious electron recombination would lower the electrons concentration, and hence lead to the decline of *J*_sc_, *η* [29] and *V*_oc_ [30]. For the optimized DSSCs based on Zn_2_SnO_4_ solid octahedrons, the *η*, *V*_oc_, *J*_sc_ and FF were 2.49%, 0.64 V, 5.65 mA cm^−2^ and 68.79%, respectively.

**Figure 2:**
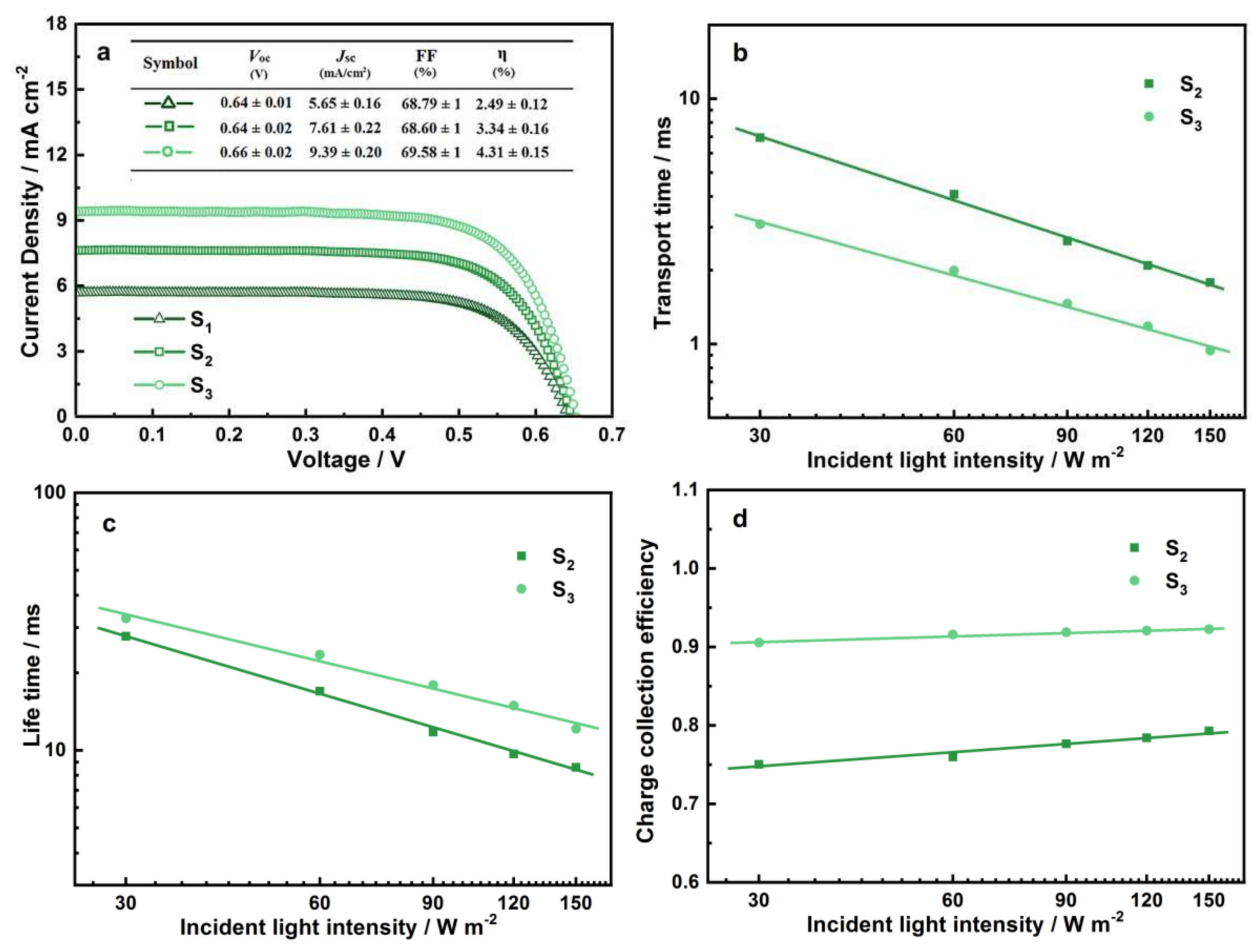
(**a**) *J−V* curves for the DSSCs based on the different electrodes; (**b**) Transport time, (**c**) Lifetime and (**d**) the charge collection efficiency of the different DSSCs.

To study the effect of the hollow structure on the photovoltaic properties of DSSCs, Zn_2_SnO_4_ hollow octahedron photoelectrodes were prepared by depositing 12 μm film on FTO glass, and photovoltaic properties were tested by constructing DSSCs. The characteristic *J*-V curves and their photovoltaic parameters are given in Figure 2a. It can be seen that the *V*_oc_ and FF of S_2_-based DSSCs were similar to those of S_1_-based DSSCs, the distinct photovoltaic behavior of S_2_ was larger *J*_sc_ (7.61 mA cm^−2^) compared to S_1_ (5.65 mA cm^−2^). The relatively high *J*_sc_ of S_2_ can be considered following the three combined effects: (1) Higher dye loading, due to larger specific surface area. The measured dye-loading amount for the S_2_ film was around 4.23 × 10^−^^7^ mol·cm^−2^ μm^−1^, and was more than that of the S_1_ film (2.87 × 10^−^^7^ mol·cm^−2^ μm^−1^). The surface area of S_2_ was measured as 37.7 m^2^ g^−1^ (Figure S4), 70.6% higher than that of S_1_ (22.1 m^2^ g^−1^). So, the larger specific surface area enabled S_2_ to adsorb more dye to improve *J*_sc_ of DSSCs. (2) Stronger light-harvesting efficiency, due to the hollow structure. The corresponding UV–vis absorption spectra of solid and hollow Zn_2_SnO_4_ octahedrons displayed a strong absorption peak in the ultraviolet region, and the absorption intensity of the latter was stronger than that of the former (Figure 1a). Because of the same crystal structure, the enhanced light absorption of S_2_ was presumably due to its hollow structure, as explained in Figure S5. Obviously, in case of Zn_2_SnO_4_ hollow octahedrons, the empty internal structure could offer more interfaces to considerably increase the chance of light reflections, leading to the more efficient utilization of the incident light and increased light-harvesting efficiency, and then more photogenerated electron-hole pairs would generate when S_2_ was under light irradiation [31,32]. (3) Efficient electrolyte diffusion into the interior of the hollow octahedrons, due to mesoporous wall. The results of N_2_ adsorption/desorption confirmed the existence of the mesoporous microstructure for Zn_2_SnO_4_ hollow octahedrons (Figure S4). The pore-size distribution indicated that the average pore size for S_2_ was about 3.6 nm, which would promote electrolytes to transport in the photoelectrode and enter the internal area of hollow octahedrons to increase the contact probability between electrolyte and electrodes to improve the *η* of DSSCs.

#### 3.2.2. Photovoltaic Characteristics of ZnSnO_3_/Zn_2_SnO_4_

To investigate the influence of the ZnSnO_3_ nanosheets on the electron transfer and recombination behaviors, the IMPS/IMVS measurements of S_2_ and S_3_ were performed. The IMPS/IMVS measurements could provide direct evidence of the electron transport time (*τ*_t_) and electron recombination time (*τ*_r_) through the equations [33]:

(2) $$\tau_{t}=1/\left(2\pi f_{t}\right)$$

(3) $$\tau_{r}=1/\left(2\pi f_{r}\right)$$

where *f*_t_ (*f*_r_) is the minimum frequency of the IMPS(IMVS) imaginary component. Here, *τ*_t_ and *τ*_r_ characterize the electron transport properties in photoanode films and recombination with redox species in the electrolyte, respectively. Hence, smaller *τ*_t_ and larger *τ*_r_ mean a faster transport rate and slower recombination rate. Examinations of Figure 2b,c clearly showed that the *τ*_t_ (*τ*_r_) of the DSSCs based on S_3_ photoanodes was shorter (longer) than that of S_2_ over light intensities from 30 to 150 W m^−2^. This finding was a consequence of the following reasons: first, the engineered interfacial design of band-structure-matched ZnSnO_3_/Zn_2_SnO_4_ composite photoanodes. A schematic illustration of the energy levels of ZnSnO_3_ and Zn_2_SnO_4_ is shown in Scheme 2b, the CB edge of ZnSnO_3_ was determined to be −0.99 V (Figure S6), which was more negative than that of Zn_2_SnO_4_ (−0.93 V, Table S1), such an energy band matching composite photoanode would serve as the “bridge” to expedite electron transport from excited ZnSnO_3_ to FTO resulting in shorter τ_t_ [34]. The band-structure-matched ZnSnO_3_/Zn_2_SnO_4_ photoanodes, meanwhile, to some extent avoid any extra internal traps, and could effectively suppress the recombination between the electron in the CB of composite photoanodes and I_3_^−^ in the electrolyte, which rationalizes the reasons for the increase in τ_r_. Second, the two-dimensional ZnSnO_3_ nanosheets in the composite photoanodes caused direct electron transport paths and were beneficial for faster electron transport. Besides, it is well known that the less trap sites there are, the slower charge recombination occurs [35]. Compared to the nanoparticles with abundant grain boundaries, ZnSnO3 nanosheets exhibited a reduced recombination loss, which can contribute to longer τ_r_. Third, interconnected contact between the ZnSnO_3_ nanosheets of neighboring octahedrons made the contact area larger and prolonged the electron transport channel of the injected electrons, so lead to fast electron transport to the back contact, thus a lower recombination rate [36]. These results verified the reasonableness of the promoting the electron transfer of the ZnSnO_3_ nanosheets in the composite film.

**Figure 3:**
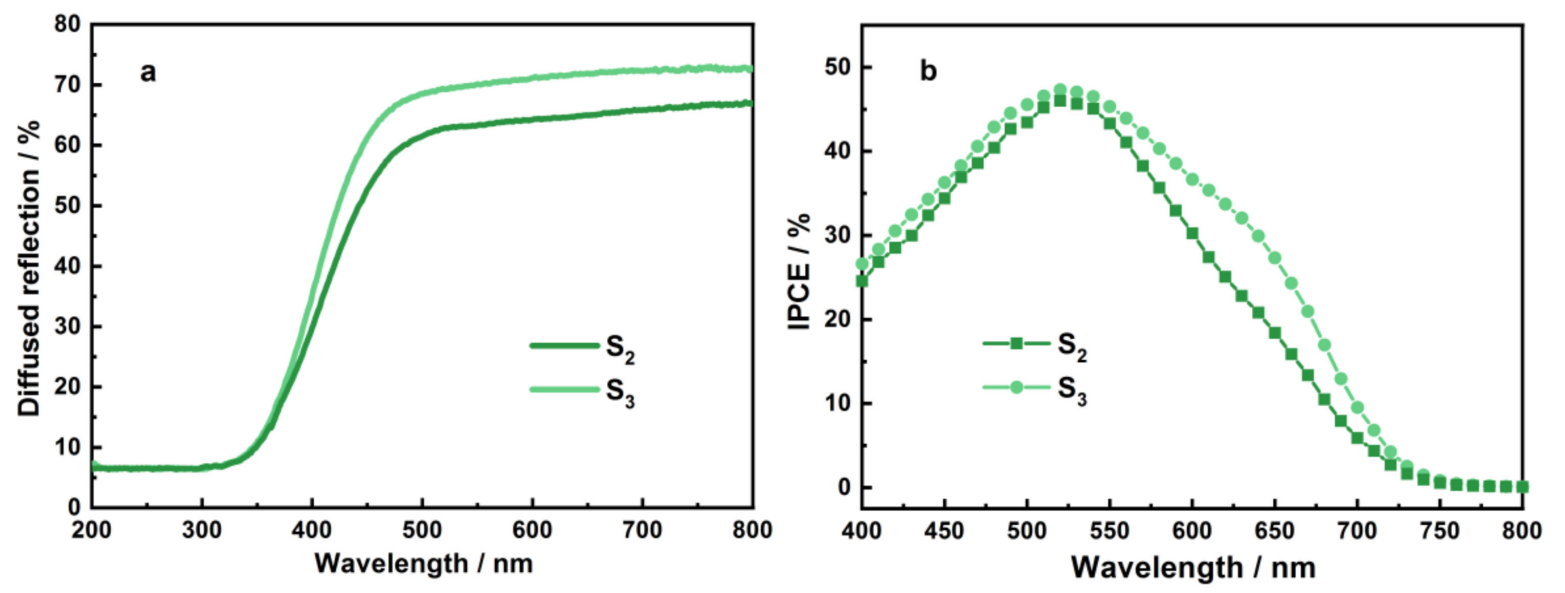
(**a**) Diffuse reflectance spectra and (**b**) IPCE spectra of the different electrodes.

**Scheme 3:**
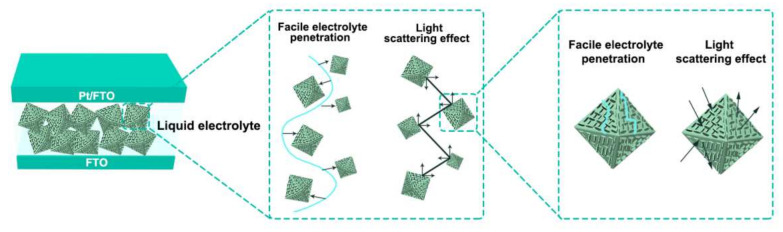
Schematic representation of (**left**) a DSSC with ZnSnO_3_/Zn_2_SnO_4_ hollow octahedron film, (**center**) facile penetration of electrolyte through the hollow octahedrons and light scattering effect by the octahedrons, and (**right**) a single octahedron showing penetration of an electrolyte through it.

To evaluate the scattering effect of the ZnSnO_3_ nanosheets, the diffuse reflection spectra of the films made from S_2_ and S_3_ were measured (Figure 3a). Compared to the Zn_2_SnO_4_ film, the composite film had higher diffuse reflection ability in the wavelength region from 380 to 800 nm, suggesting that the vertical ZnSnO_3_ nanosheets would lead to more significant incident light scatter within the composite films. A possible explanation can be that S_3_, owing to the surface ZnSnO_3_ nanosheets, can scatter the incident light to all directions, as schematically illustrated in Scheme 3. Thereby, the composite film utilized the incident light to a higher degree than that in the case of S_2_. At the same time, this scattering capacity could provide more chances for the N719 molecules on the composite area to trap photons, and then turn into electrons [26]. Otherwise, compared with S_2_, the absorption edge (~390 nm) of S_3_ exhibited red shift (Figure 1a), implying more holes and electrons can be generated after ZnSnO_3_ incorporation, which probably was attributed to the interaction between ZnSnO_3_ and Zn_2_SnO_4_. These results confirmed the positive impact of the ZnSnO_3_ nanosheets in the composite film on the light harvesting efficiency. 

To evaluate the beneficial impact of the addition of the ZnSnO_3_ nanosheets on the photovoltaic conversion properties, the composite was applied as photoanodes of DSSCs. Four ZnSnO_3_/Zn_2_SnO_4_ DSSCs with the same thickness (~12 µm) were fabricated with various sensitization durations. The *η* gradually increased with the sensitization time and reached the maximum at 4 h (Table S4). Figure 2a shows the *J–V* curves of the DSSCs based on the optimized composite photoanode film DSSCs. Compared with DSSCs based on S_2_, the addition of ZnSnO_3_ nanosheets to Zn_2_SnO_4_ cells resulted in considerable improvement in *V*_oc,_ *J*_sc_ and FF, so the DSSCs with S_3_ exhibited an enhanced *η* (4.31%) value by around 27.9% as compared with S_2_ DSSCs (3.34%). It can be seen that the *V*_oc_ of the S_3_ (0.66 V) was higher than that obtained from the S_2_ (0.64 V). The *V*_oc_ is determined by the chemical potential of the electrolyte and the Fermi level of the semiconductor oxide [37]. As seen in Scheme 2a, the S_3_ showed a more negative *E*_fb_ value, so the difference between the Fermi level of S_3_ and the redox potential of I^−^/I_3_^−^ would be larger, and the corresponding *V*_oc_ will be higher. On the other hand, through the analysis of the IMVS and IMPS dates above, the introduction of ZnSnO_3_ nanosheets in the Zn_2_SnO_4_ film can inhibit electron recombination and prolong electron lifetime, which could increase the *V*_oc_. So, the enhanced *V*_oc_ with ZnSnO_3_ nanosheets added should offer the combined impact of the elevated CB edge and the suppressed interfacial charge recombination. 

The *J_sc_* can be estimated using the following formula [38]:

(4) $$J_{sc}=qI_{0}\eta_{lh}\eta_{inj}\eta_{cc}$$

where *q* and *I*_0_ are the elementary charge and light flux. Hence, the *J*_sc_ is directly proportional to the light harvesting efficiency (*η*_lh_), electron injection efficiency (*η*_inj_) and electron collection efficiency (*η*_cc_) of DSSCs. First, the *η*_lh_ is determined by both the dye loading amount and/or the light scattering capability [39]. The absorption quantity of N719 on S_3_ with the surface area of 41.3 m^−2^ g^−1^ (4.49 × 10^−7^ mol·cm^−2^ μm^−1^) was slightly larger than that of S_2_ (4.23 × 10^−7^ mol·cm^−2^ μm^−1^). Meanwhile, as discussed above, the addition of the ZnSnO_3_ nanosheets favored enhanced light-harvesting efficiency and originated from light scattering and the red shift of the absorption edge. To further obtain the detailed information on the light harvest of the DSSCs, the IPCE measurements were performed, as shown in Figure 3b. The IPCE value of the S_3_-cell was higher than that of the S_2_-cell in whole wavelength range from 400 to 800 nm, indicating that the quantum efficiency of the S_3_-cell was greater than that of the S_2_-cell in the visible-light wavelength region [40]. The higher IPCE values for the S_3_-cell at short wavelengths reflected on its stronger dye-loading capacity. In the longer wavelength region from 600 to 750 nm, the increased IPCE values of the S_3_-cell could be explained by the enhanced light scattering efficiency, which promotes the light harvesting of the N719 dye in this region. Further, the IPCE measurements also validated the estimated *J*_sc_ values (Figure S7) and correlated well with those obtained from the J-V curves (Figure 2a). Those studies indicated superior light harvesting efficiency of S_3_ films compared to S_2_ films. Second, φ_inj_ is the probability of electron injection from the dye-excited state into the CB of the semiconductor. The CB was moving upward in the S_3_ film, which meant that charge injection from LUMO of N719 dye to the CB of S_3_ would be limited, thus reducing φ_inj_. Third, the *η*_cc_ can be estimated according to *τ*_t_ and *τ*_r_ [33].

(5) $$\eta_{\mathrm{cc}}=1-\;\tau_{t}/\tau_{r}$$

Figure 2d showed the *η*_cc_ of the S_3_-cell was superior to the S_2_-cell under various light intensities, which is attributed to the incorporation of ZnSnO_3_ nanosheets enhancing the electron transfer. In summary, although the φ_inj_ values would proportionally decrease, the S_3_ showed an increased *J*_sc_ because the *η*_lh_ and *η*_cc_ were raised more significantly than the decrease in φ_inj_.

A comparison of photovoltaic performances between our assembled DSSCs and previously reported DSSCs is summarized in Table S5, including *J*_sc_, *V*_oc_, FF and *η*. It can be seen that, the *η* of the DSSC based on ZnSnO_3_/Zn_2_SnO_4_ hollow octahedrons was not higher than that of Ba^2+^ ion doped Zn_2_SnO_4_ nanocrystalline [41], the cause was probably that the photo sensitizer was a mixture including N719 dye and D131 dye instead of only N719. The DSSC based on ZnSnO_3_/Zn_2_SnO_4_ hollow octahedrons showed superior photovoltaic performances compared to metallic silver decorated Zn_2_SnO_4_ spheres [42], Au inlaid Zn_2_SnO_4_ /SnO_2_ hollow rounded cubes [43], the binary metal oxide MgO passivation layers modified Zn_2_SnO_4_ nanoparticles [44], and ZnO/Zn_2_SnO_4_ core shell photoanodes [45]. In addition, the higher FF showed the advantage of ZnSnO_3_/Zn_2_SnO_4_ hollow octahedrons in practical application [46].

### 3.3. Photocatalytic Performance

**Figure 4:**
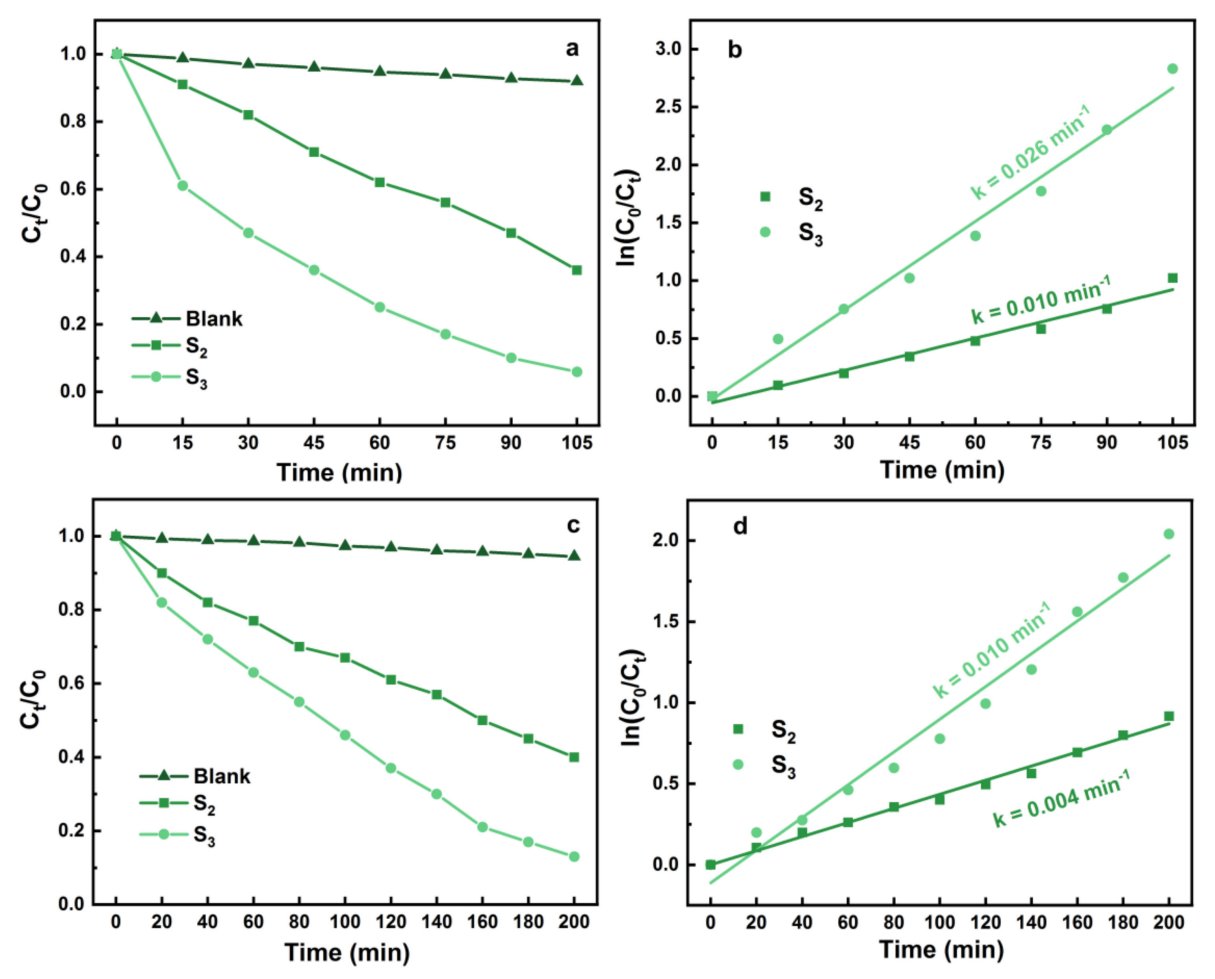
Removal efficiency of (**a**) CIP and (**c**) Cr(VI); the first−order kinetics of (**b**) CIP and (**d**) Cr(VI) by different photocatalysts in single system.

**Figure 5:**
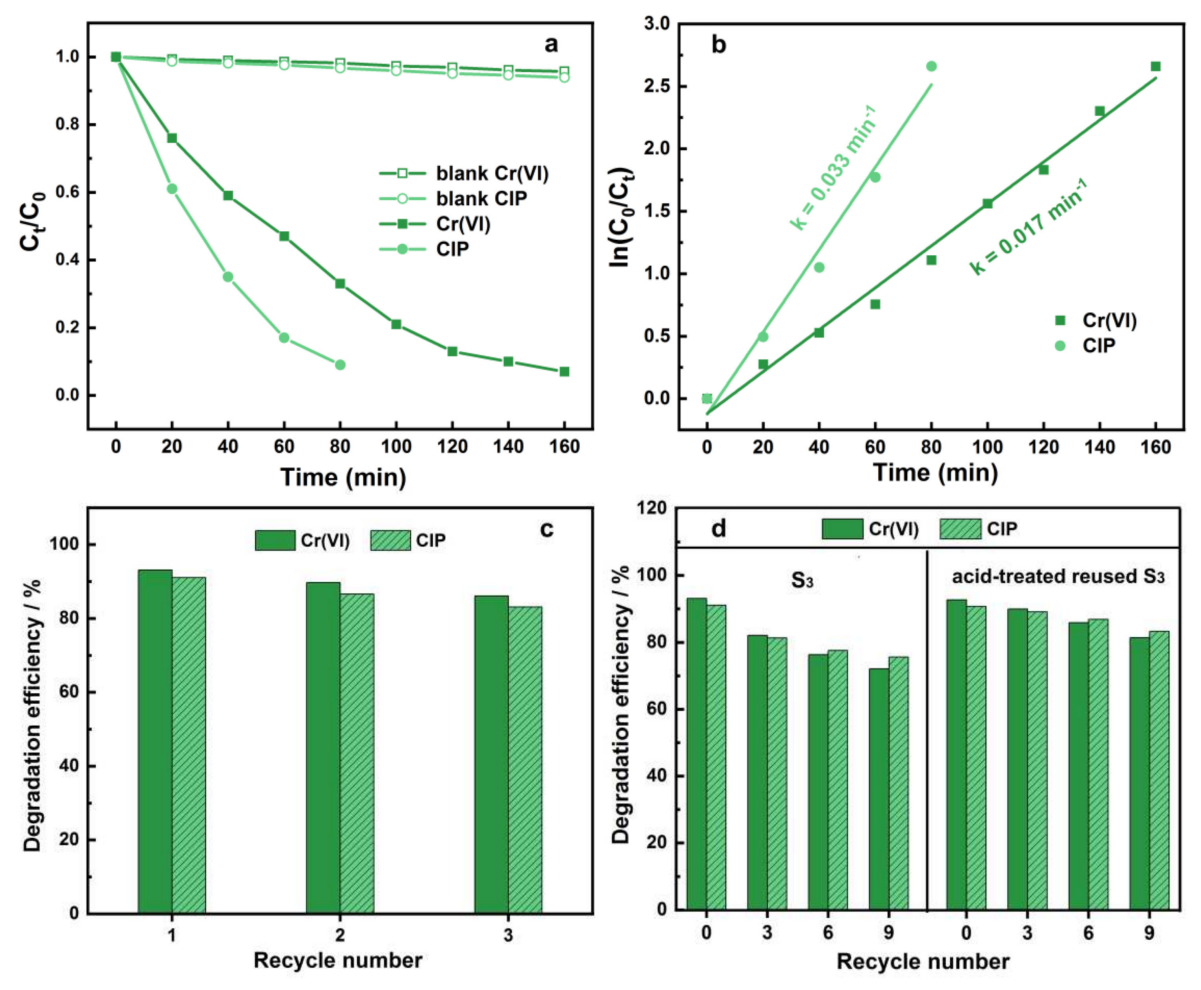
(**a**) Removal efficiency of CIP and Cr(VI) in the mixed solutions by S_3_ sample; (**b**) the corresponding first-order kinetics and rate constants; (**c**) reusable degradation activity of S_3_ for three cycles; (**d**) simultaneous removal of CIP and Cr(VI) in the presence of S_3_ with and without acid treatment for nine cycles.

In order to further demonstrate the beneficial effect of the ZnSnO_3_ nanosheets on photocatalytic activity, S_2_ and S_3_ were used to perform photocatalytic removal of CIP and Cr(VI) in single and co-existing systems under simulated sunlight. A blank control experiment was carried out to confirm that CIP would hardly self-degrade under the condition of no photocatalyst (Figure 4a). Initially, only 64.3% of CIP was degraded by S_2_ within 105 min of light irradiation. With the addition of ZnSnO_3_, the catalyzed degradation efficiency of the composites reached 94.3% within same irradiation time length. As shown in Figure 4b, the photodegradation of CIP catalyzed by S_2_ and S_3_ followed a pseudo-first-order rate law (Table S6), ln (C_0_/C_t_) = *k*t, where *k* is the degradation rate constant, C_0_ and C_t_ are the concentrations of the pollutant solution before light irradiation and at irradiation time *t*, respectively. The *k* values of S_2_ and S_3_ were 0.010 and 0.026 min^−1^ (Figure 4b), respectively, and the photodegradation rate constant of S_3_ was around 2.6 times higher than that of S_2_. The solution of Cr(VI) was also treated to investigate the photoreduction performance of photocatalyst. As shown in Figure 4c, the reduction of Cr(VI) was almost negligible in the absence of the photocatalyst, and the Cr(VI) concentration was decreased by the processes of simulated sunlight. In 200 min, 60.5% and 87.1% photoreduction efficiency was observed using S_2_ and S_3_, respectively. The kinetic constant 0.010 min^−1^ of S_3_ was numerically about 2.50 times as high as S_2_ at 0.004 min^−1^ (Figure 4d). All these showed, compared with the S_2_ sample, that the S_3_ photocatalyst displayed stronger removal efficiency under illumination, which verified that the addition of ZnSnO_3_ nanosheets effectively enhanced system photocatalytic performance. For further research, the simultaneous photocatalytic CIP oxidation and Cr(VI) reduction over S_3_ were performed in the co-existing systems containing CIP (10 mg/L)/K_2_Cr_2_O_7_ (50 mg/L). S_3_ only removed a small amount of CIP and Cr(VI) under a no-light condition, which showed no simulated sunlight response (Figure 5a). Compared with the photodegradation results in Figure 4 and Figure 5b, the higher CIP removal performance presented after the introduction of Cr(VI) in the system, and the *k* value increased from 0.026 min^−1^ without Cr(VI) to 0.033 min^−^^1^ with co-existence of Cr(VI). Meanwhile, the removal rate of Cr(VI) increased with co-existence of CIP, and the k value of S_3_ in the mixed solution was 1.70 times as high as in the absence of CIP. The results of the experiment suggested the synergistic effect between photocatalytic CIP oxidation and Cr(VI) reduction.

The quenching experiments were conducted to further explore the removal mechanism of Cr(VI) and CIP over S_3_ (Figure S8). In general, ammonium oxalate (AO), isopropyl alcohol (IPA), dimethyl sulfoxide (DMSO) and benzoquinone (BQ) were used to quench the active species photogenerated holes (*h*^+^), hydroxyl radicals (OH), photogenerated electrons (*e*^−^) and superoxide radicals (O_2_^−^), respectively [47]. It could be observed that the photodegradation efficiencies of CIP decreased evidently with the addition of AO and BQ to the co-existing system, reflecting that *h*^+^ and O_2_^−^ had a significant effect in the removal reaction of CIP over S_3_. In contrast, when the IPA was introduced into this system, there was only a negligible suppression, indicating the influence of ·OH could be ignored. Therefore, *h*^+^ and O_2_^−^ were the predominant reactive species in the CIP photocatalytic degradation process. Meanwhile, the photoreduction efficiencies of Cr(VI) obviously decreased from 92.9 to 47.6% with the addition of DMSO, it could be deduced that Cr(VI) was reduced by *e*^−^ and the photogenerated electrons served as the main reaction species of the photocatalytic reduction of Cr(VI).

Based on the above results and discussion, a photocatalytic mechanism was proposed (Scheme 2b). When the light irradiated on the photocatalyst, the ZnSnO_3_/Zn_2_SnO_4_ composite could be excited by the photon, and equivalent energy would generate the electron-hole pairs. The conduction band of ZnSnO_3_ (−0.99 V) was more negative than that of the hollow Zn_2_SnO_4_ (−0.93 V), the photogenerated electrons would easily inject from the former into the latter through the heterostructure between ZnSnO_3_ and Zn_2_SnO_4_. Therefore, the photogenerated electron-hole pairs were spatially separated by this heterostructure to restrain the recombination of charges. Cr(VI) was a strong oxidant and could serve as an electron acceptor to consume these generated electrons. Because the E_CB_ of Zn_2_SnO_4_ was more negative than the O_2_/·O_2_^−^ potential (−0.33 eV) [48], and the electrons would react with the dissolved O_2_ to produce O_2_^−^. Meanwhile, the valence band of Zn_2_SnO_4_ (2.66 V) was more positive than that of ZnSnO_3_ (2.23 V), and the holes would migrate simultaneously from VB of Zn_2_SnO_4_ to ZnSnO_3_ in the opposite direction, whereas, h^+^ at VB of ZnSnO_3_ was not more positive than the H_2_O/·OH (2.40 eV) [48], and not enough to oxidize H_2_O to ·OH. Hence, the O_2_^−^ and h^+^ acted as the main oxidants to decompose CIP into small molecules. So, in this co-existing system, the faster photoreduction rate of Cr(VI) and photodegradation efficiency of CIP might be due to the synchronized actions of the CIP as an electron donor and Cr(VI) as an electron acceptor. The oxidation process of CIP and the reduction process of Cr(VI) consumed the photo-excited holes and electrons, respectively. These two processes effectively promoted the separation of photogenerated electrons and holes, and resulted in much more electrons for Cr(VI) reduction and holes for CIP oxidation, and the recombination of photogenerated carriers would be prohibited so as to enhance the photocatalytic efficiency [47].

To evaluate the reusable ability of S_3_ in the co-existing systems, the recycling experiment was conducted under the same conditions. As shown in Figure 5c, after the three cycles of operation, the removal efficiencies changed from 92.7% to 86.1% for Cr(VI) and 91.1 to 83.2% for CIP, and the photocatalytic performance showed less than 9% loss, which confirmed that the ZnSnO_3_/Zn_2_SnO_4_ composite possessed outstanding reusability. To further investigate the repeatable removal performance, the number of repeated uses was increased from three to nine, and the photocatalytic efficiency decreased to 71.8% for Cr(VI) and 75.3% for CIP. Borrowed from the previous method [49], after three consecutive cycles, S_3_ was cleaned with 0.1 mM HCl once to remove the adsorbed pollutant molecules, it was found that S_3_ with the acid-treatment preserved 88.5 and 89.2% of the initial efficiency for Cr(VI) and CIP in the ninth cycle, and greater than 15.6 and 10.4% photocatalytic efficiency without the acid-treatment, respectively (Figure 5d). So, the hierarchical ZnSnO_3_/Zn_2_SnO_4_ porous hollow octahedrons could be applied in the simultaneous removal of antibiotic and heavy metal pollutants from contaminated water for environmental protection.

## 4. Conclusions

To summarize, hierarchical ZnSnO_3_/Zn_2_SnO_4_ porous hollow octahedrons were designed by structure and composition evolution. According to IMVS/IMPS, reflectance spectra, IPCE and Mott-Schottky plots measurements, ZnSnO_3_ nanosheets introduced on the surface of hollow Zn_2_SnO_4_ octahedrons could provide a direct pathway for electron transport, act as light-scattering centers to improve light harvesting, and induce the flat-band potential to shift negatively. As a result, as-prepared ZnSnO_3_/Zn_2_SnO_4_ hollow octahedrons could enhance the overall photovoltaic conversion efficiency by 73.1% compared to that of the Zn_2_SnO_4_ solid octahedrons. Meanwhile, the composite also exhibited higher removal activity of Cr(VI) and CIP in single and co-existing systems under simulated sunlight irradiation. In addition, the composite photocatalyst showed good durability and stability in a co-existing system, suggesting its potential practical applications in the simultaneous removal of heavy metal and antibiotic pollutants from contaminated water.

## Data Availability

The data supporting the findings of this study are available by reasonable request to nylzd@nynu.edu.cn.

## Supplementary Materials

The following supporting information can be downloaded at: https://www.mdpi.com/article/10.3390/nano12122124/s1, Figure S1: Schematic of the dye-sensitized solar cell; Figure S2: SEM images of (a) S_1_, (b) S_2_, (d) S_3_ and (f) ZnSnO_3_ nanosheets; TEM images of (c) S_2_ and (e) S_3_. Inset: the scale bar is 50 nm in (d); Figure S3: XRD patterns of (a) S_1_, (b) S_2_ and (c) S_3_; Figure S4: N_2_ adsorption/desorption isotherms: (a) S_2_ and (b) S_3_; the inset is corresponding pore size distribution curves; Figure S5: Schematic illustration of light reflections and refractions in S_2_; Figure S6: ZnSnO_3_ nanosheets: (a) UV-vis absorption spectra; (b) Mott-Schottky plots; Inset of (a): the corresponding Tauc’s curves; Figure S7: The estimated current density by integrating the IPCE spectrum; Figure S8: Controlling experiments using different radical scavengers for the removal of CIP and Cr(VI); Table S1. The calculated results of the samples about band gap energy (E_g_), flat band potential (E_fb_), E_CB_ and E_VB_; Table S2: Detailed photovoltaic parameters of solid Zn_2_SnO_4_-based DSSCs with film thicknesses of 10 μm and various dye-loading times; Table S3: Detailed photovoltaic parameters of DSSCs based on the Zn_2_SnO_4_ photoanode with different film thicknesses; Table S4: Detailed photovoltaic parameters of ZnSnO_3_/Zn_2_SnO_4_-based DSSCs with film thicknesses of 12 μm and various dye-loading times; Table S5: Comparison of the photovoltaic performance of ZnSnO_3_/Zn_2_SnO_4_ hollow octahedrons against previously reported DSSCs; Table S6: The fitting results about photocatalytic removal of the pollutants over the different samples.
