# Supplementary material for: In Situ Decoration of ZnSnO3 Nanosheets on the Surface of Hollow Zn2SnO4 Octahedrons for Enhanced Solar Energy Application
Source: Nanomaterials (Basel). 2022 Jun 20;12(12):2124. doi: 10.3390/nano12122124 (PMC9230825; doi:10.3390/nano12122124)
Supplement: Supplementary file 1 [file nanomaterials-12-02124-s001.zip › nanomaterials-1768885-supplementary.pdf]

Supplementary material

In Situ Decoration of $ZnSnO_3$ Nanosheets on the Surface of Hollow Zn_2SnO_4 Octahedrons for Enhanced Solar Energy Application

Zhengdao Li [1,2,*], Kecheng Liu [1], Ruixue Sun [1], Chuanyun Yang [1,2] and Xiaodi Liu [1,*]

1 Chemistry and Pharmaceutical Engineering College, Nanyang Normal University, Nanyang 473061, China; iukecheng2006@126.com (K.L.); ruixuesun@126.com (R.S.); ycy_chemistry@163.com (C.Y.)
2 Engineering Technology-Research Center of Henan Province for Solar Catalysis, Nanyang Normal University, Nanyang 473061, China
* Correspondence: nylzd@nynu.edu.cn (Z.L.); liuxiaodiny@126.com (X.L.); Tel: +86-377-63-513-735 (Z.L.)

Materials Synthesis

Preparation of solid Zn_2SnO_4 octahedrons (sample 1: S_1): 0.26 g of zinc acetate dehydrate ($ZnAc_2 \cdot 2H_2O$), 0.22 g of chloride pentahydrate ($SnCl_4 \cdot 5H_2O$), 0.29 g of sodium hydroxide (NaOH) and 0.25 g of hexadecyl trimethyl ammonium bromide (CTAB) were mixed in 30 mL of deionized water. Then, the mixture was put into an autoclave (50 mL) and heated for 6 hours at 200 °C. The precipitate was collected and washed many times with water and dried at 60 °C.

Preparation of hollow Zn_2SnO_4 octahedrons (sample 2: S_2): 0.5 g of Zn_2SnO_4 solid octahedrons was dispersed in 1 M nitric acid solution (50 mL) under ultrasonic condition for 5 minutes, whereafter, the powder was bathed in acid solution at room temperature for 3 hours. This product was washed after centrifuging and dried at 60 °C in air for 6 hours.

Preparation of $ZnSnO_3$ nanosheets: 0.29 g of zinc nitrate hexahydrate ($Zn(NO_3)_2 \cdot 6H_2O$), 0.27 g of sodium sulfite trihydrate ($Na_2SnO_3 \cdot 3H_2O$), 10 mL of ethanol and 20 mL of H_2O were added to an autoclave (50 mL) with agitation at room temperature. A solvothermal process proceeded for 20 hours at 200 °C, and the product was collected after washing and drying at 80 °C.

Hierarchical $ZnSnO_3/Zn_2SnO_4$ hollow octahedrons (sample 3: S_3) were prepared in two steps: the first step was the preparation of $ZnSnO_3$ sol, 4.38 g of $ZnAc_2 \cdot 2H_2O$ was dissolved in 100 mL of ethylene glycol monomethyl ether ($HOCH_2CH_2OCH_3$) to form white suspension under vigorous stirring conditions. An amount of 2 mL of monoethanolamine ($HOCH_2CH_2NH_2$) and 7.0 g of $SnCl_4 \cdot 5H_2O$ were added into the mixture, stirring constantly for 1 hour. This transparent and colorless liquid was heated to reflux for 4 hours at 80 °C to obtain transparent, homogeneous and pale-yellow sol. The as-prepared Zn_2SnO_4 hollow octahedrons were immersed in the $ZnSnO_3$ sol for 12 hours to gain the $ZnSnO_3$ seed layer on the surface of S_2. The second step was the preparation of $ZnSnO_3$ sheets on the surface of Zn_2SnO_4 hollow octahedrons. An amount of 0.15 g of $ZnSnO_3$-seeded S_2 was dumped into a mixture consisting of 0.29 g of $Zn(NO_3)_2 \cdot 6H_2O$, 0.27 g of $Na_2SnO_3 \cdot 3H_2O$, 10 mL of ethanol and 20 mL of H_2O. Next, the obtained slurry was put into an autoclave (50 mL) and the solvothermal reaction continued for 20 hours at 200 °C. The obtained precipitate was centrifuged, washed, and the dried solid powder was calcined for 3 hours at 600 °C in air with a heating rate of 5 °C.

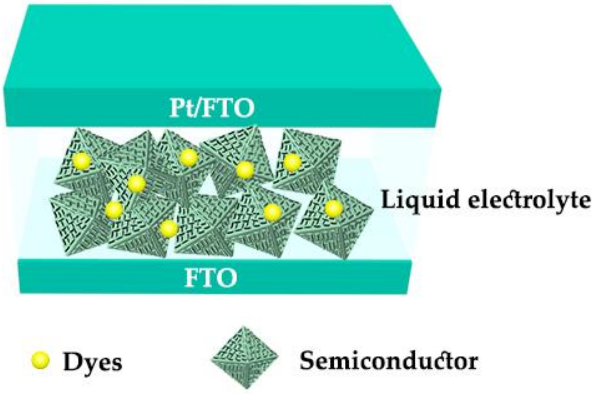

Figure S1. Schematic of the dye-sensitized solar cell.

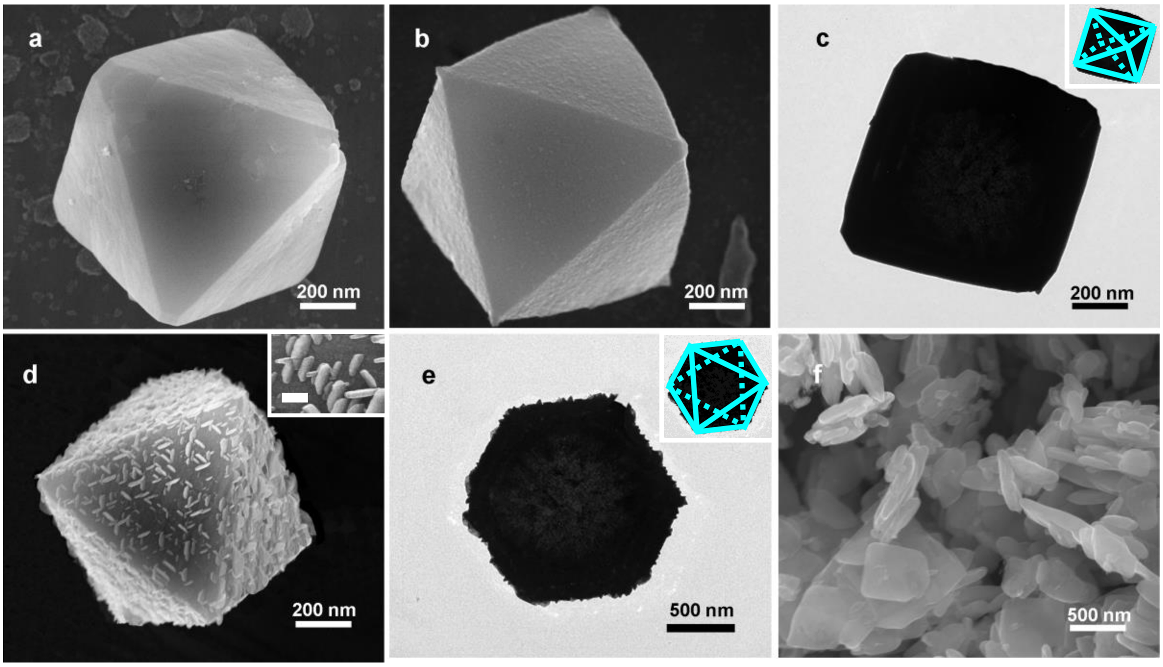

Figure S2. SEM images of (**a**) S_1, (**b**) S_2, (**d**) S_3 and (**f**) $ZnSnO_3$ nanosheets; TEM images of (**c**) S_2 and (**e**) S_3. Inset: the scale bar is 50 nm in (**d**).

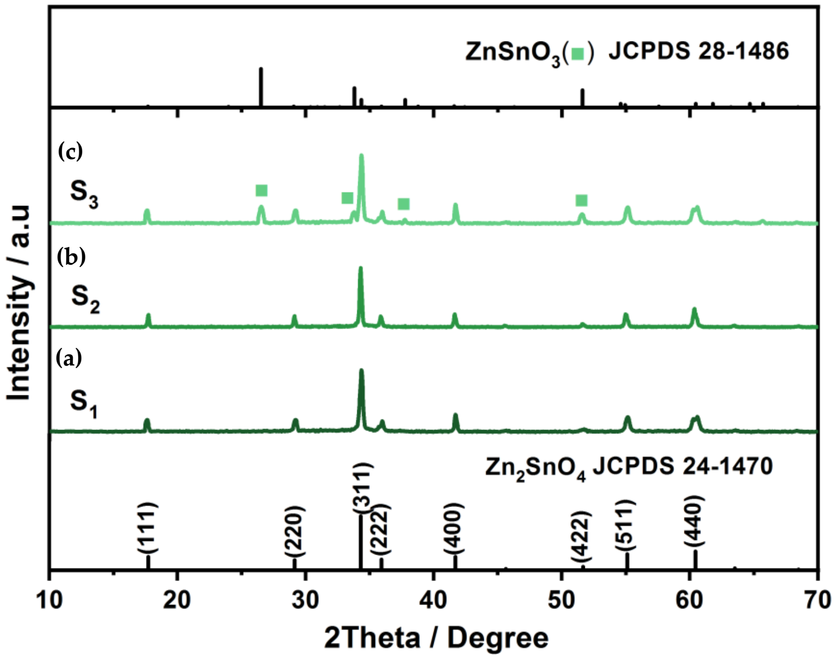

Figure S3. XRD patterns of (**a**) S_1, (**b**) S_2 and (**c**) S_3.

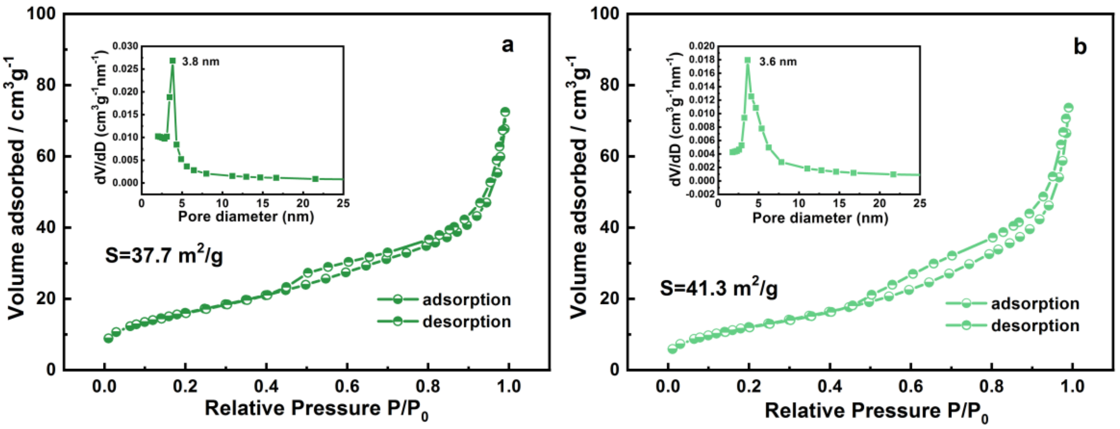

Figure S4. N_2 adsorption/desorption isotherms: (**a**) S_2 and (**b**) S_3; the inset is corresponding pore size distribution curves.

The surface area and pore size were measured through the Barett–Joyner–Halenda (BJH) and Brunauer–Emmett–Teller (BET) measurement methods (Quantachrome NOVA 4200e). The BET surface areas of S_2 and S_3 were 37.7 and 41.3 $m^2\ g^{-1}$, respectively (Figure S3). The pore size distribution curves in the inset of Figure S3 revealed that S2 and S3 had a narrow pore-size distribution, and the mesoporous existed in two sample judged by the distribution center size of 3.8 nm and 3.6 nm [12].

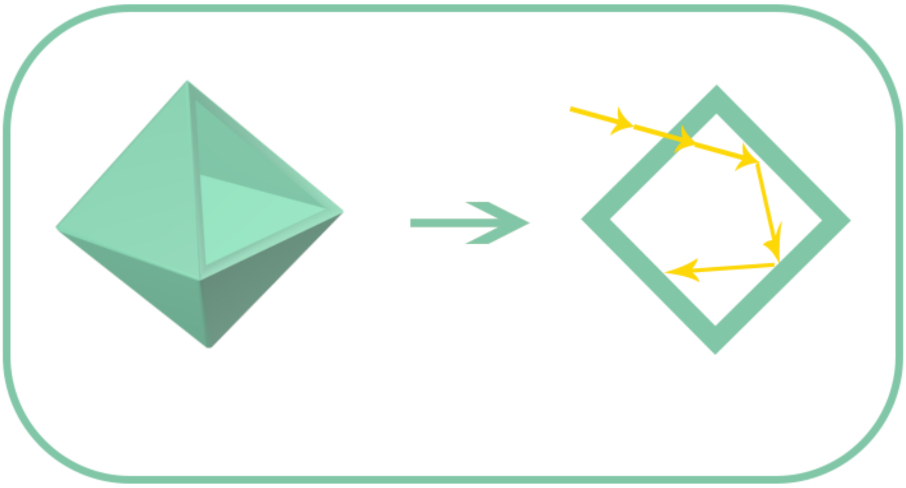

Figure S5. Schematic illustration of light reflections and refractions in S2.

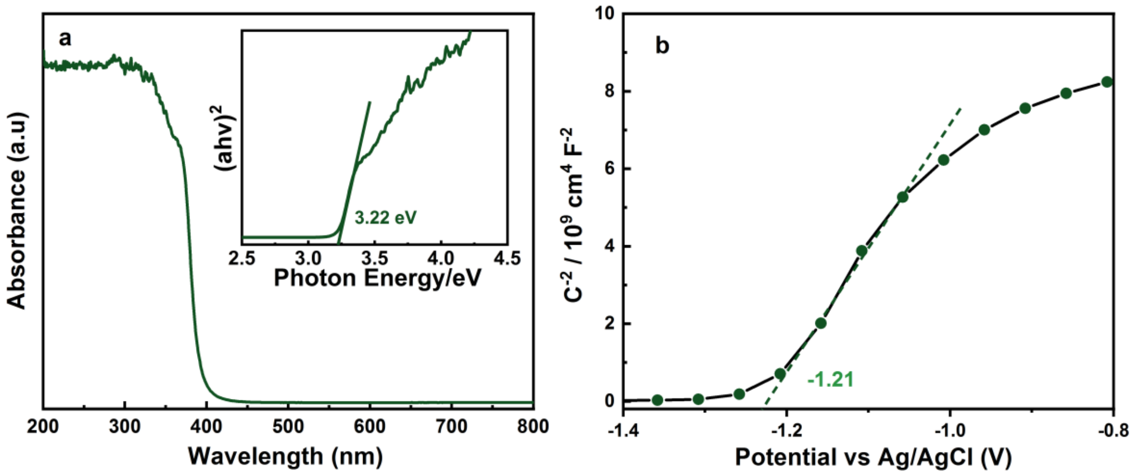

Figure S6. $ZnSnO_3$ nanosheets: (**a**) UV-vis absorption spectra; (**b**) Mott-Schottky plots; Inset of (a): the corresponding Tauc's curves.

Based on the results of Figure S5, the E_g of the $ZnSnO_3$ nanosheet was estimated to be around 3.22 eV, and the E_{fb} was calculated to be -1.21 V (vs. Ag/AgCl, pH=7). Then, the conduction band (CB) potentials could be determined to be -0.99V (vs NHE, pH=7). According to the formula ($E_{VB} = E_{CB} + E_g$), the valence band (VB) potential of the $ZnSnO_3$ nanosheet was calculated to be 2.23 V.

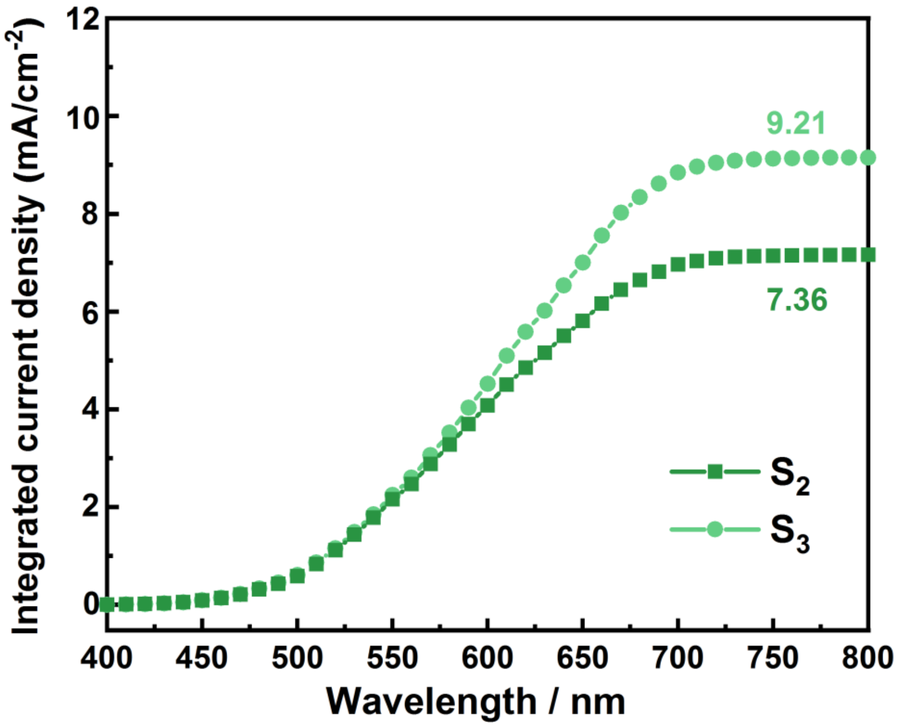

Figure S7. The estimated current density by integrating the IPCE spectrum.

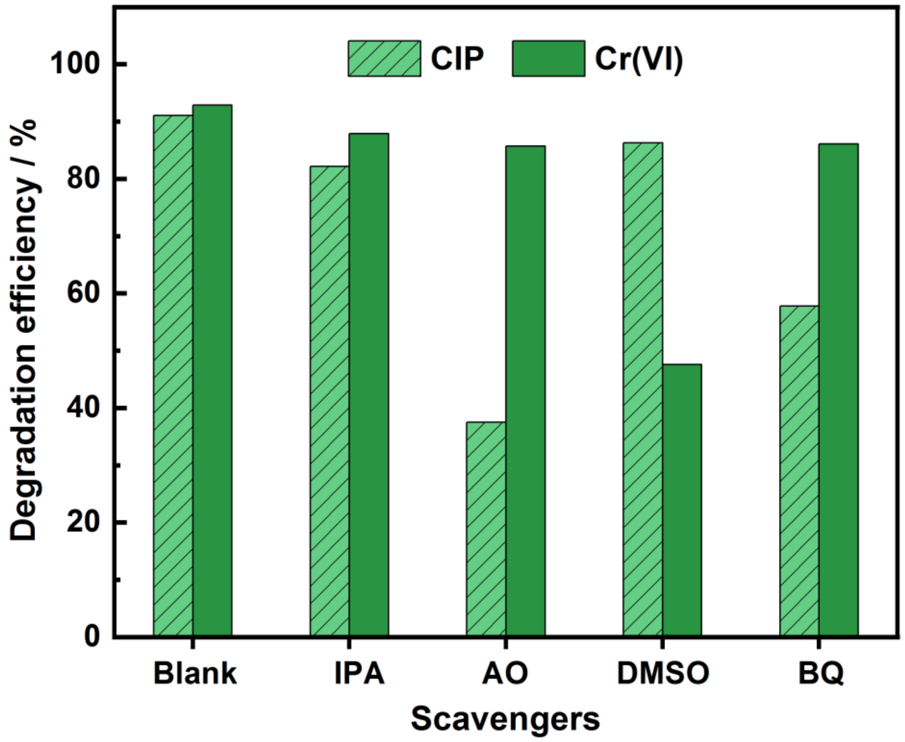

Figure S8. Controlling experiments using different radical scavengers for the removal of CIP and Cr(VI).

Table S1. The calculated results of the samples about band gap energy (E_g), flat band potential (E_{fb}), E_{CB} and E_{VB}.

Samples	Eg (eV)	E_{fb} (*vs* Ag/AgCl)	E_{CB} (*vs* NHE)	E_{VB} (*vs* NHE)
**S_1**	3.83	-1.15	-0.93	2.90
**S_2**	3.59	-1.15	-0.93	2.66
**S_3**	3.43	-1.29	-1.07	2.36
**$ZnSnO_3$ nanosheet**	3.22	-1.21	-0.99	2.23

Table S2. Detailed photovoltaic parameters of solid Zn_2SnO_4-based DSSCs with film thicknesses of 10 μm and various dye-loading times.

Time	V_{oc} (mV)	J_{sc} (mA cm^{-2})	FF	η (%)
**2** hours	637 ± 9	3.77 ± 0.11	67.84 ± 1	1.63 ± 0.07
**4** hours	643 ± 7	4.82 ± 0.07	68.12 ± 1	2.11 ± 0.05
**6** hours	644 ±10	5.32 ± 0.11	68.71 ± 1	2.35 ± 0.11
**8** hours	643 ± 8	4.29 ± 0.14	66.33 ± 1	1.83 ± 0.10

Table S3. Detailed photovoltaic parameters of DSSCs based on the Zn_2SnO_4 photoanode with different film thicknesses.

Film thickness	V_{oc} (mV)	J_{sc} (mA cm^{-2})	FF	η (%)
**6 μm**	651 ± 9	4.17 ± 0.09	70.03 ± 1	1.90 ± 0.08
**9 μm**	646 ±11	4.91 ± 0.13	68.71 ± 1	2.18 ± 0.09
**12 μm**	643 ± 10	5.65 ± 0.16	68.79 ± 1	2.49 ± 0.12
**15 μm**	639 ± 12	4.36 ± 0.17	65.93 ± 1	1.83 ± 0.11
**18 μm**	576 ±10	3.25 ± 0.15	63.12 ± 1	1.18 ± 0.07

Table S4. Detailed photovoltaic parameters of $ZnSnO_3/Zn_2SnO_4$-based DSSCs with film thicknesses of 12 μm and various dye-loading times.

Time	V_{oc} (mV)	J_{sc} (mA cm^{-2})	FF	η (%)
**3** hours	660±4	7.13±0.09	67.13±1	3.16±0.07
**4** hours	663±2	9.39±0.20	69.58±1	4.31±0.15
**5** hours	661±3	8.22±0.13	67.18±1	3.65±0.09
**6** hours	657±5	7.06±0.15	65.23±1	3.03±0.08

Table S5. Comparison of the photovoltaic performance of $ZnSnO_3/Zn_2SnO_4$ hollow octahedrons against previously reported DSSCs.

Samples	V_{oc} (V)	J_{sc} (mA cm^{-2})	FF	η (%)	Ref.
Ba^{2+}/ Zn_2SnO_4	0.679	9.83	0.739	4.94	[41]
Ag/Zn_2SnO_4	0.76	3.83	0.55	1.618	[42]
Au/Zn_2SnO_4/SnO_2	0.60	5.88	57.7%	2.04	[43]
MgO/Zn_2SnO_4	0.66	8.59	0.51	2.90	[44]
ZnO/Zn_2SnO_4	0.61	17.8	0.31	3.36	[45]
$ZnSnO_3$/Zn_2SnO_4	0.66	9.39	69.58%	4.31	This work

Table S6. The fitting results about photocatalytic removal of the pollutants over the different samples.

Samples	System	Pollutants	Fitting formula	*k*	R^2
**S_2**	single	CIP	Y=0.00931X-0.05523	0.010	0.9817
**S_3**	single	CIP	Y= 0.02561X-0.02426	0.026	0.9835
**S_2**	single	Cr(VI)	Y=0.00435X-1.764*10^{-4}	0.004	0.9821
**S_3**	single	Cr(VI)	Y= 0.01011X-0.11242	0.010	0.9833
**S_3**	co-existing	CIP	Y=0.03298X-0.12417	0.032	0.9813
	co-existing	Cr(VI)	Y=0.01678X-0.11827	0.017	0.9807
